# Endoscopic Management of Polyps Arising From Diverticula

**DOI:** 10.14309/crj.0000000000000779

**Published:** 2022-05-17

**Authors:** Mohammed Shakhatreh, Grigoriy L. Rapoport, Asif Zamir

**Affiliations:** 1 UTRGV School of Medicine–Doctor's Hospital at Renaissance Gastroenterology Fellowship Program, Edinburg, TX; 2 Renaissance Gastroenterology, Edinburg, TX

## CASE REPORT

Colon polyps arising from diverticula are rare with only a few cases reported to date.^1^ Most colon diverticula are considered “false diverticula” and are composed mainly of mucosa and submucosa that protrude through the muscularis externa layer and are covered by the serosa. Because of this, endoscopic resection of such polyps carries a high risk of perforation and management has traditionally been with surgical intervention, which increases the risk of complications. In this series, we present 2 cases of successful excision of an adenomatous polyp located within the lumen of a diverticulum using endoscopic mucosal resection (EMR).

### Case 1

**Figure 1.**
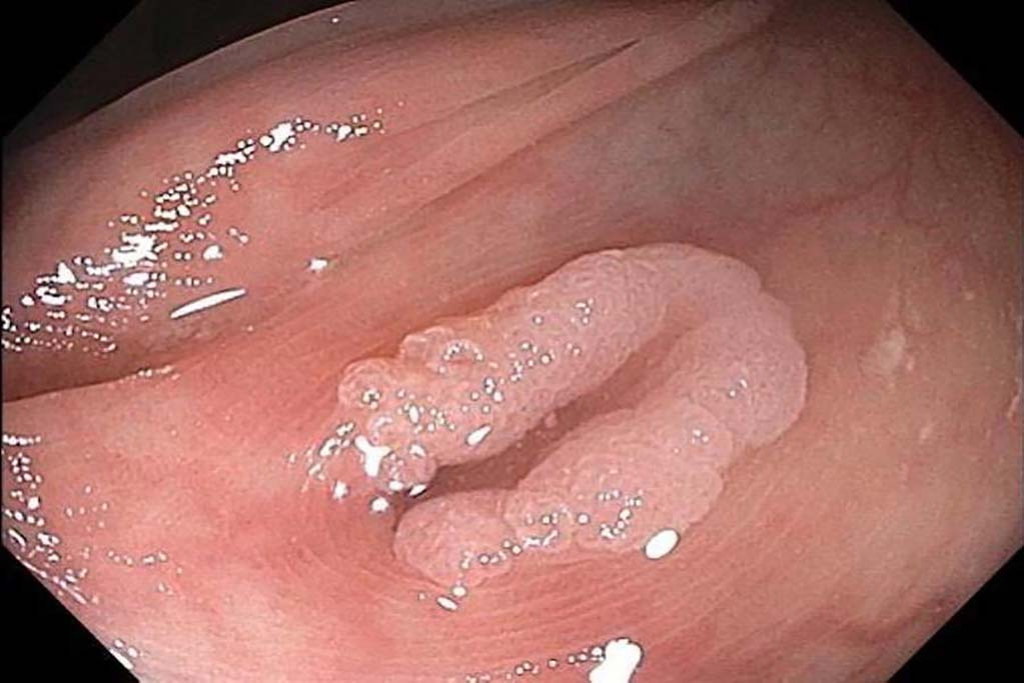
Polyp arising from the diverticulum.

**Figure 2.**
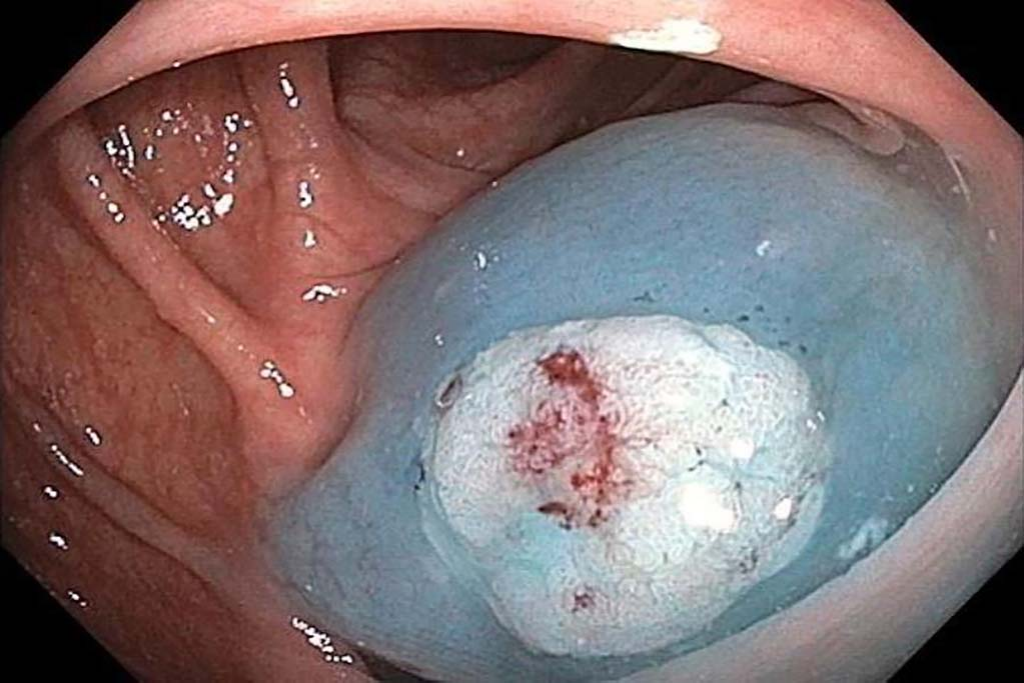
Polyp raised with ORISE gel.

**Figure 3.**
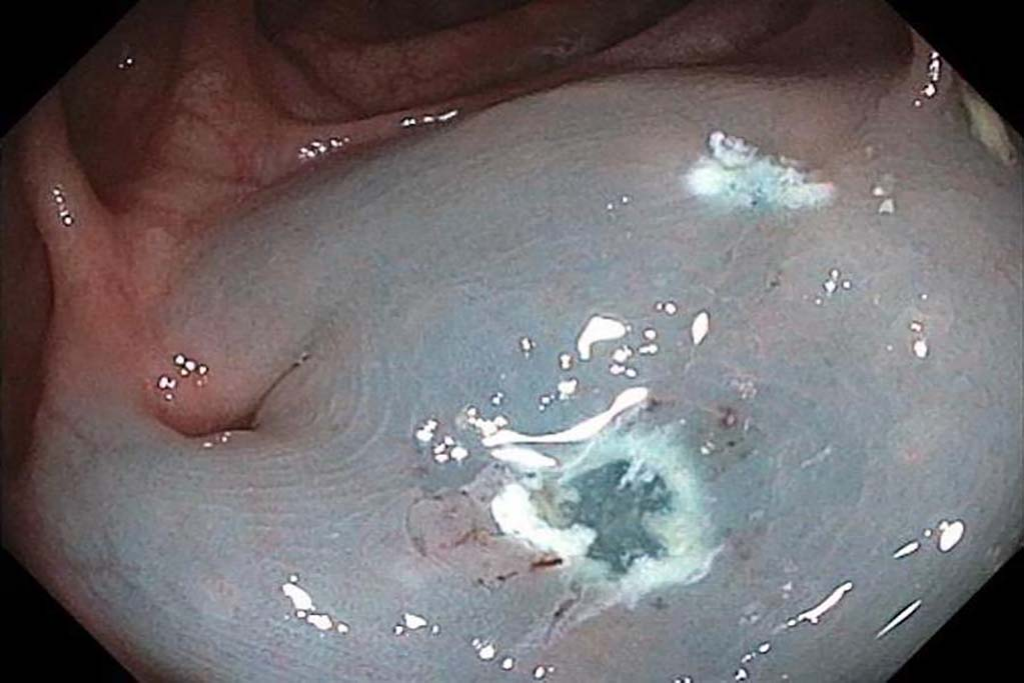
Polypectomy site.

**Figure 4.**
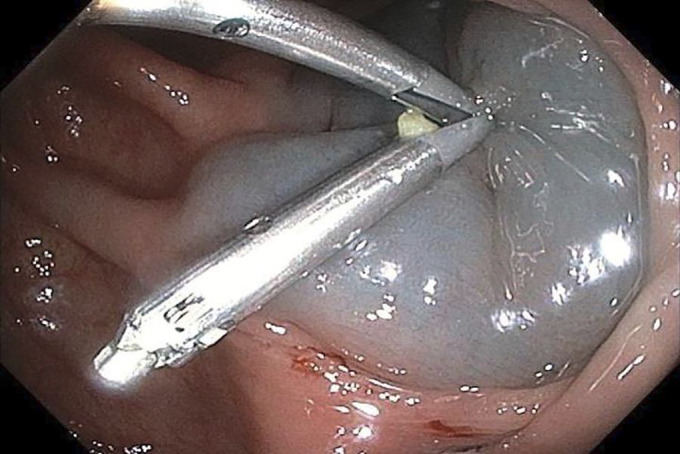
Endoclip deployment after resection.

A 73-year-old woman presented for a surveillance colonoscopy. During the procedure, a 1-cm polyp was found arising from within a diverticulum in the proximal transverse colon (Figure 1). The area was carefully inspected, and a decision was made to remove the polyp using hot snare EMR. ORISE gel was injected within the diverticulum, raising the polyp out of the diverticulum, making it accessible for EMR (Figure 2). Then, a 10-mm Captivator II snare (Boston Scientific, Marlborough, MA) was used to remove the polyp by the 1-piece snare polypectomy technique using a blend cut mode (Figure 3). Two Resolution Clips (Boston Scientific) were prophylactically deployed at the site of polypectomy (Figure 4). Pathology confirmed tubular adenoma. The patient did not experience any early or delayed complications.

### Case 2

A 72-year-old woman presented for a surveillance colonoscopy. During the procedure, an 8-mm, flat polyp was found arising from a diverticulum in the hepatic flexure. After careful inspection, a decision was made to remove the polyp using hot snare EMR. We injected the site of the polyp with ORISE gel, raising the polyp out, making it accessible for EMR. A 10-mm Captivator II snare (Boston Scientific) was used to remove the polyp by the 1-piece snare polypectomy technique using the blend cut mode. Two Resolution Clips (Boston Scientific) were used prophylactically at the site of polypectomy. Pathology revealed tubular adenoma. The patient did not experience any early or delayed complications.

Colon polyps that arise from diverticula are very rare.^1^ When detected, endoscopic removal carries a high risk of perforation. EMR has revolutionized endoscopic removal of advanced polyps. Other techniques described in a few case reports include endoscopic band ligation and the use of over-the-scope clips with full-thickness colon resection.^2–5^ To the best of our knowledge, this is the first series of cases that demonstrate successful removal of diverticular polyps using EMR without any complications, but more cases are needed to validate this technique as a standard of care. Informed consent could not be obtained from the patient despite several attempts. All identifying information has been removed from this case report to protect patient privacy.

## DISCLOSURES

Author contributions: M. Shakhatreh and G. Rapoport reviewed the literature and wrote and edited the article. A. Zamir provided the images, approved and revised the final article for intellectual content, and is the article guarantor.

Financial disclosure: None to report.

Previous presentation: Case 1 was presented at the American College of Gastroenterology Annual Scientific Meeting; October 23-28, 2020; virtual meeting.

Informed consent could not be obtained for this case report. All identifying information has been removed.
